# Molecularly informed preoperative and response-adapted management of colorectal cancer: evidence, decision boundaries, and emerging directions

**DOI:** 10.3389/fonc.2026.1894870

**Published:** 2026-09-09

**Authors:** Wenjing Hao, Yan Liu, Haidong Cheng, Mingxing Hou

**Affiliations:** 1 Inner Mongolia Medical University, Hohhot, China; 2 Affiliated Hospital of Inner Mongolia Medical University, Hohhot, China

**Keywords:** colorectal cancer, colorectal liver metastases, immunotherapy, molecular profiling, neoadjuvant therapy, organ preservation, total neoadjuvant therapy, watch-and-wait

## Abstract

Treatment delivered before surgery in colorectal cancer now encompasses several clinically distinct strategies rather than one uniform pathway. These include selective neoadjuvant chemotherapy for radiologically high-risk colon cancer, total neoadjuvant therapy (TNT) and response-adapted organ preservation in rectal cancer, immunotherapy for mismatch repair-deficient or microsatellite instability-high (dMMR/MSI-H) disease, and perioperative or conversion treatment for colorectal liver metastases (CRLM). This Review separates these settings by treatment intent, expected endpoint, likelihood of surgery, and evidence maturity. MMR/MSI has direct treatment relevance in localized disease; RAS/BRAF status and primary tumor sidedness mainly inform metastatic and conversion strategies; HER2-directed treatment remains supported principally by advanced-disease data; and circulating tumor DNA (ctDNA), radiomics, and other dynamic tools remain adjunctive or investigational for most preoperative decisions. Quantitative results from pivotal trials are summarized alongside practical decision boundaries, including clinical complete response assessment, watch-and-wait surveillance, CRLM resectability reassessment, and stopping rules. The central message is deliberately cautious: moving treatment earlier is useful only when the clinical scenario, therapeutic aim, evidence level, reassessment plan, and salvage pathway are defined before treatment begins.

## 1 Introduction

Colorectal cancer (CRC) remains a major cause of cancer morbidity and mortality worldwide, but the decisions facing multidisciplinary teams are no longer determined by stage alone (1, 2). Treatment selection now integrates the anatomical site of the primary tumor, the probability of margin-negative surgery, the presence of synchronous or metachronous metastases, tumor biology, patient fitness, and the feasibility of high-quality surveillance. These considerations become pivotal when systemic therapy is delivered before surgery: the preoperative window can improve systemic treatment delivery, but it can also delay definitive local control when the indication is poorly chosen.

In this Review, the broader term “preoperative and response-adapted management” is used to avoid treating unlike strategies as interchangeable. Neoadjuvant systemic therapy refers to systemic treatment given for a resectable primary tumor with surgery still planned. TNT denotes delivery of all intended systemic chemotherapy and pelvic radiotherapy before rectal surgery and is therefore not purely “systemic” therapy. Perioperative therapy spans treatment before and after a planned operation. Conversion therapy is reserved for initially unresectable CRLM treated with the explicit aim of achieving R0 resection or another no-evidence-of-disease (NED) local strategy; it is not synonymous with conventional neoadjuvant therapy. Response-adapted organ preservation refers to a post-treatment decision pathway in which surgery may be deferred after a clinical complete response (cCR), whereas watch-and-wait (W&W) is the active surveillance program used to implement non-operative management (NOM).

The rationale for moving systemic therapy earlier remains strong. Administering treatment before surgery may help target occult micrometastatic disease, improve patient tolerance and completion of combination regimens, shrink locally advanced tumors, increase the likelihood of achieving an R0 resection, and create additional opportunities for organ preservation. At the same time, this strategy is not without risk. Imaging can overestimate disease extent, potentially exposing some patients to unnecessary treatment when surgery alone might have been sufficient. Preoperative therapy may also lead to cumulative toxicity, delay potentially curative surgery or miss resection windows in patients with CRLM, and encourage premature adoption of biomarkers and therapies that have not yet been fully validated. Consequently, the central issue is no longer simply whether earlier systemic therapy is feasible. Rather, it is: who needs intensification, and who can safely receive less?

When decisions are made before surgery, CRC should not be approached as a uniform disease. A low rectal tumor threatening the mesorectal fascia, a radiologically defined T4 colon cancer, a dMMR colon cancer, a dMMR rectal tumor showing clinical complete response after PD-1 blockade, and liver-limited metastatic disease stratified by RAS/BRAF status each represent biologically and clinically distinct situations that require different therapeutic reasoning. Randomized trials and clinical guidelines have clarified several important areas, particularly the role of TNT in LARC and the growing importance of molecular profiling before treatment selection. Yet significant disagreements continue over treatment intensity, organ preservation, conversion therapy, and dynamic monitoring (3–7).

This Review therefore organizes the evidence within a biologically informed, scenario-based framework. Rather than providing an exhaustive catalog of regimens, we ask five recurring questions: What is the disease state? What is the treatment intent? Which factor triggers escalation or de-escalation? When should response or resectability be reassessed? What evidence boundary limits routine use? The article addresses locally advanced colon cancer (LACC), locally advanced rectal cancer (LARC), dMMR/MSI-H disease, CRLM, and emerging molecular or dynamic tools. Across sections, three evidence categories are used consistently: Tier 1, guideline-supported or high-level evidence applicable when trial eligibility is met; Tier 2, prospective but non-definitive evidence supporting selective or conditional use in experienced multidisciplinary settings; and Tier 3, investigational or emerging evidence that should not direct routine preoperative care. These categories are an author-developed interpretive decision framework, not a formal GRADE assessment or a substitute for guideline grading. Each tier applies to a defined clinical decision in a specified setting rather than to a biomarker, drug, or assay in isolation.

## 2 Literature search and evidence selection

The original narrative search used PubMed/MEDLINE and the China National Knowledge Infrastructure (CNKI), supplemented by reference-list screening of guidelines, landmark trials, prospective studies, and high-quality reviews. Because the original platform export was not retained, retrospective numerical flow counts were not reconstructed. For this revision, PubMed/MEDLINE was updated from database inception through 14 August 2026. Representative syntax combined controlled vocabulary and title/abstract terms for colorectal, colon, or rectal cancer with “neoadjuvant,” “preoperative,” “perioperative,” “total neoadjuvant,” “watch and wait,” “organ preservation,” “conversion therapy,” “liver metastases,” “immunotherapy,” “ctDNA,” or “radiomics”. Separate topic modules were run for LACC, rectal TNT/W&W, dMMR or pMMR immunotherapy, CRLM, and biomarkers. CNKI verification searches used the corresponding Chinese subject-field concepts and were executed from database inception through 16 August 2026 in the Academic Journals/All Journals collection. The five modules returned 106 records for LACC, 135 for rectal TNT/W&W, 30 for immunotherapy, 144 for CRLM, and 32 for biomarkers. All 447 citation records were exported; exact-citation de-duplication across modules yielded 442 unique records. All unique records underwent title/citation-level relevance screening. Records considered potentially capable of changing a clinical decision boundary were checked against full reports or primary sources, and no quantitative conclusion was derived from citation-only records. These counts describe the revision-period verification search only and were not used to reconstruct the unavailable historical search flow. Evidence eligible for the core synthesis was limited to full reports available in English or Chinese. The complete representative Boolean strings, search dates, platform settings, 442-record citation registry, and update-screening record are provided in **Supplementary File 1**.

Eligible evidence included guidelines or consensus statements, randomized trials, prospective phase II/III studies, large registries or observational cohorts, systematic reviews, and clinically relevant translational studies that directly informed a prespecified decision scenario. Conference abstracts without a full report, duplicate reports lacking additional follow-up, studies without a preoperative or response-adapted decision link, and advanced-disease studies used to imply routine perioperative efficacy were excluded from the core synthesis. WH led title/citation-level screening, full-source verification, evidence extraction, and drafting; YL conducted targeted supplementary literature searches during revision and organized the retrieved evidence and citation records; HC provided senior intellectual guidance and critical review of the revised conceptual framework and interpretation; and MH supervised the clinical framework and reviewed the resulting interpretation. When evidence conflicted, mature randomized outcomes and guideline recommendations were prioritized, negative trials and later follow-up were retained, and differences in population, endpoint, treatment intent, and follow-up were made explicit rather than pooled qualitatively. No formal risk-of-bias instrument or meta-analysis was performed because this is a structured narrative Review.

## 3 Scope, taxonomy, and decision framework

**Figure 1:**
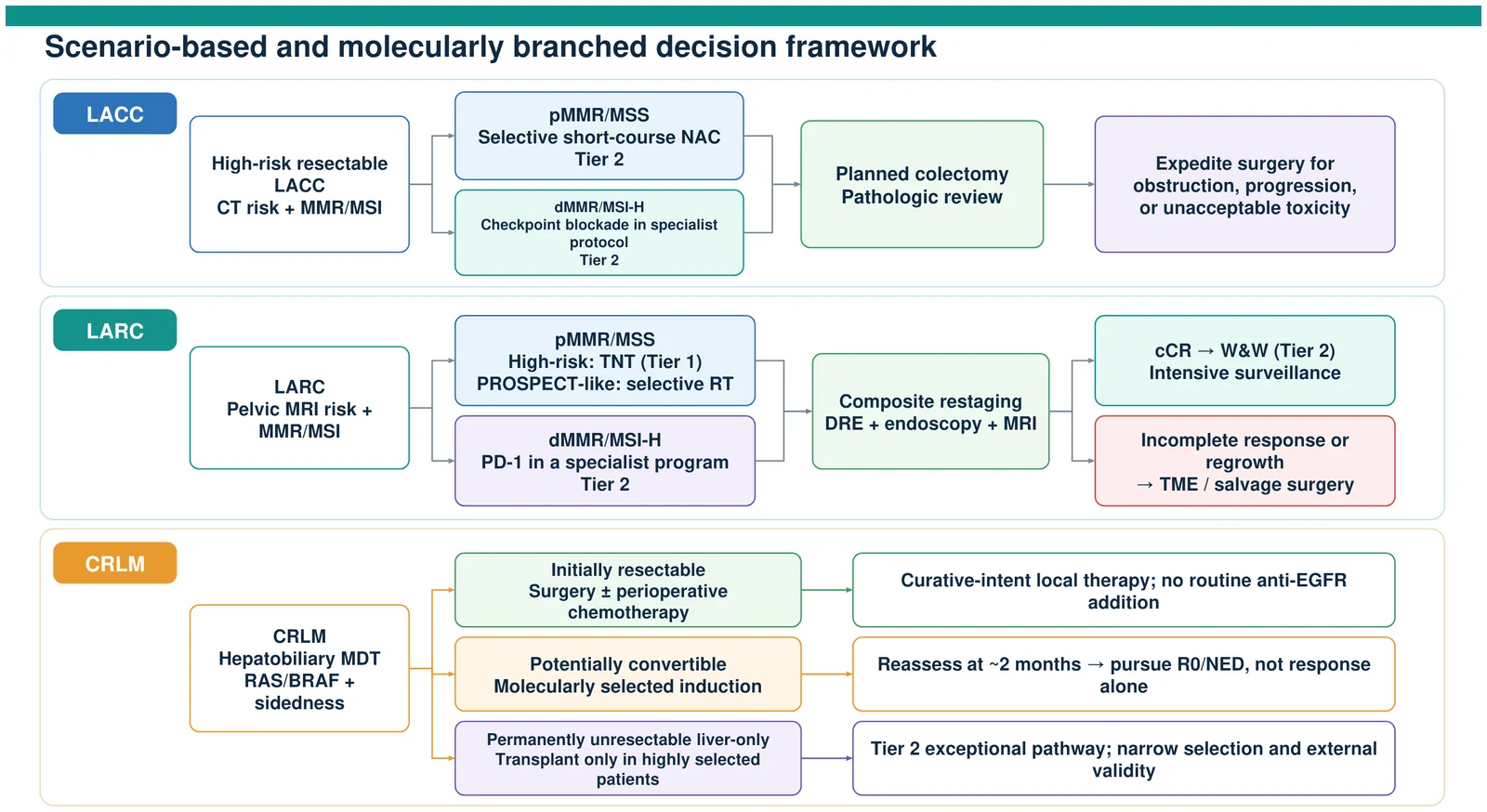
Scenario-based and molecularly branched decision framework for preoperative and response-adapted management of colorectal cancer. **Figure 1** separates LACC, LARC, and CRLM before treatment selection. Within the LACC and LARC pathways, pMMR/MSS and dMMR/MSI-H are mutually exclusive branches rather than sequential treatments. LACC pathways retain planned colectomy; LARC pathways converge on composite restaging and then diverge to TME or W&W after a documented cCR. CRLM is divided at baseline into initially resectable, potentially convertible, and permanently unresectable liver-only categories, each with an independent aim, reassessment plan, and stopping boundary. Tier labels apply to the stated clinical decision and represent an author-developed interpretive framework rather than formal GRADE assessment; colors distinguish clinical pathways, not evidence strength. cCR, clinical complete response; CRLM, colorectal liver metastases; CRT, chemoradiotherapy; CT, computed tomography; dMMR/MSI-H, mismatch repair-deficient/microsatellite instability-high; DRE, digital rectal examination; LACC, locally advanced colon cancer; LARC, locally advanced rectal cancer; MDT, multidisciplinary team; MRF/CRM, mesorectal fascia/circumferential resection margin; NED, no evidence of disease; NOM, non-operative management; pMMR/MSS, mismatch repair-proficient/microsatellite stable; R0, microscopically margin-negative resection; TNT, total neoadjuvant therapy; TME, total mesorectal excision; W&W, watch-and-wait.

Preoperative planning begins by assigning the patient to the correct clinical scenario, because the therapeutic aim and evidentiary threshold differ. In resectable LACC, a neoadjuvant strategy is intended to improve margin clearance or address systemic risk without compromising timely colectomy. In LARC, TNT combines systemic and radiation components and may prioritize distant control, local control, treatment completion, or organ preservation. In dMMR/MSI-H disease, immunotherapy sensitivity can alter the sequence, but a rectal cCR and a colon pathological response lead to different decisions. In CRLM, resectable perioperative treatment, conversion therapy for initially unresectable disease, and transplantation for selected permanently unresectable liver-only disease are separate pathways. **Figure 1** makes these distinctions explicit.

Careful baseline staging is equally important, as treatment decisions increasingly rely on detailed anatomical and biological information obtained before surgery. In rectal cancer, pelvic MRI remains central to local staging by defining tumor height, T stage, extramural vascular invasion (EMVI), nodal involvement, and involvement of the mesorectal fascia or circumferential resection margin (MRF/CRM) (8–10). These findings help estimate the risk of local failure and guide decisions regarding TNT or selective radiotherapy. In colon cancer, CT features suggesting T4 disease, extensive nodal involvement, threatened margins, or impending obstruction may justify neoadjuvant chemotherapy, although selection criteria remain less standardized than in rectal cancer. For CRLM, planning requires multidisciplinary assessment, including liver MRI, contrast-enhanced CT, evaluation of extrahepatic disease, and discussion within an experienced hepatobiliary team.

Molecular profiling should now be obtained early rather than treated as a late-line add-on. MMR/MSI status can alter the entire treatment sequence in localized and locally advanced disease. RAS and BRAF status influence anti-EGFR and anti-VEGF choices in conversion strategies. HER2 amplification, NTRK/RET fusions, POLE/POLD1 alterations, and BRAF V600E mutations may guide treatment in advanced disease and future trial selection (3, 6, 7, 11–17). A modern preoperative pathway should therefore avoid fixing the treatment sequence before the most relevant molecular data are available.

Treatment goals should be explicit before therapy begins. A patient with high-risk LARC may be treated to reduce distant relapse risk and maximize local control. A patient with lower-risk rectal cancer may be considered for de-escalation when the likelihood of margin-negative surgery is high and response is favorable. A patient with dMMR/MSI-H rectal cancer may be considered for immunotherapy with the possibility, but not the guarantee, of non-operative management (NOM). A patient with initially unresectable CRLM may be treated to create a narrow surgical window that must be captured promptly rather than to continue systemic therapy until maximal radiographic response.

The multidisciplinary team (MDT) is therefore not a procedural formality. It should define baseline resectability, risk category, molecular status, treatment aim, anticipated reassessment time point, criteria for changing strategy, and surveillance feasibility. In CRLM, this is particularly important because resectability is dynamic and depends on surgical expertise, future liver remnant, lesion distribution, treatment response, liver toxicity, and patient fitness. Contemporary trials such as CAIRO5 demonstrate the importance of central multidisciplinary assessment and repeated reassessment during induction therapy (18).

**Figure 2:**
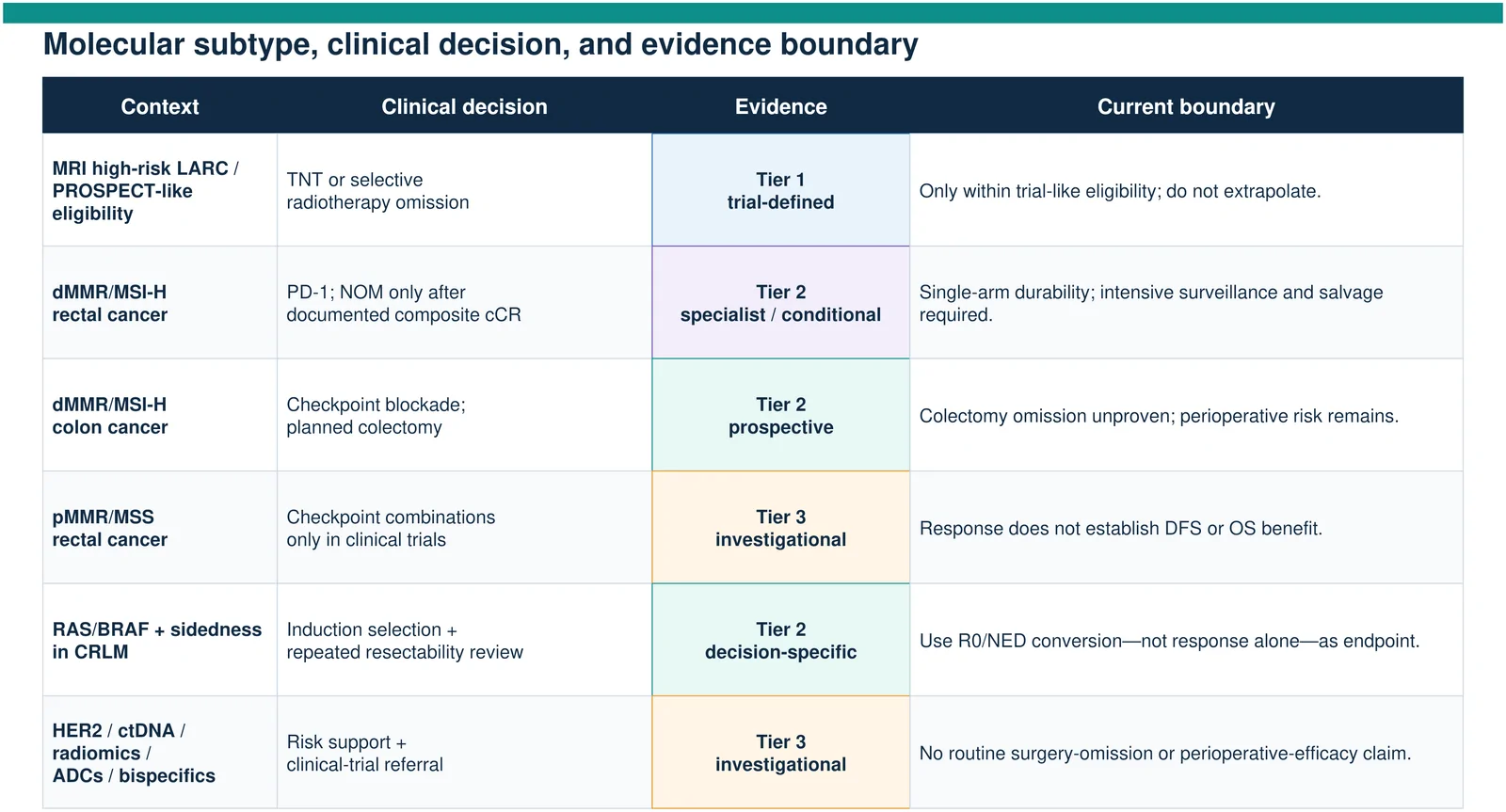
Molecular subtype, clinical decision, and evidence-boundary matrix. Legend: **Figure 2** maps each anatomical or molecular context to a specific clinical decision, evidence tier, and current applicability boundary. Tier 1 includes trial-defined escalation or de-escalation when eligibility is met. Tier 2 includes, dMMR colon immunotherapy followed by planned colectomy, dMMR rectal response-adapted NOM after documented cCR, and CRLM conversion in specialist settings. Tier 3 includes pMMR/MSS checkpoint combinations, ctDNA-guided surgery omission, radiomics, and perioperative HER2-, ADC-, or bispecific-antibody strategies. The tiers are decision-specific and author-developed, not formal GRADE ratings of a biomarker or drug. Boundary statements distinguish pathological or clinical response from survival benefit and advanced-disease activity from proven perioperative efficacy. ADC, antibody-drug conjugate; cCR, clinical complete response; CRLM, colorectal liver metastases; dMMR/MSI-H, mismatch repair-deficient/microsatellite instability-high; LACC, locally advanced colon cancer; LARC, locally advanced rectal cancer; MDT, multidisciplinary team; MRD, minimal residual disease; MRF/CRM, mesorectal fascia/circumferential resection margin; NED, no evidence of disease; NOM, non-operative management; pMMR/MSS, mismatch repair-proficient/microsatellite stable; TNT, total neoadjuvant therapy; W&W, watch-and-wait.

The framework is completed by scheduled response reassessment. Preoperative treatment should be long enough for the expected biological effect but should not continue after a curative local opportunity has emerged or when progression, unacceptable toxicity, or loss of surgical fitness changes the balance. Reassessment may integrate cross-sectional imaging, pelvic MRI, endoscopy, digital rectal examination (DRE), carcinoembryonic antigen, and pathology review. ctDNA/MRD or radiomic tools may be supportive in research or selected contexts, but they do not supersede validated anatomical and clinical assessments. **Figure 1** separates the anatomical pathways and their decision triggers; **Figure 2** encodes Tier 1, Tier 2, and Tier 3 evidence and the associated escalation, de-escalation, or research boundary.

## 4 Neoadjuvant chemotherapy in locally advanced colon cancer

The biological rationale for neoadjuvant chemotherapy in colon cancer is plausible, but routine use is not established. Upfront oncological resection followed by pathology-directed adjuvant therapy remains appropriate for most nonmetastatic cases. A preoperative approach is most defensible when high-quality computed tomography suggests T4 disease, a threatened margin, bulky nodal disease, or likely multivisceral resection and when obstruction or perforation is not imminent. Radiologic overstaging and delayed surgery remain real harms, and MMR/MSI status should be available before chemotherapy is selected.

FOxTROT provides the most mature randomized support for a selective short-course approach. Among 1,053 patients with radiologically staged T3-4, N0-2, M0 colon cancer, six weeks of preoperative oxaliplatin-fluoropyrimidine chemotherapy increased histologically complete resection from 89% to 94% and reduced two-year residual or recurrent disease from 21.5% to 16.9% (rate ratio 0.72, 95% CI 0.54-0.98), without increasing perioperative morbidity. However, 4.3% developed obstruction requiring expedited surgery, and little benefit was apparent in dMMR tumors. The trial supports carefully selected pMMR/MSS disease; it does not justify universal neoadjuvant chemotherapy (19).

OPTICAL and NeoCol sharpen this boundary. In OPTICAL, 744 patients were included in the modified intention-to-treat analysis: three-year disease-free survival was 82.1% with perioperative chemotherapy and 77.5% with upfront surgery (hazard ratio 0.74, 95% CI 0.54-1.03), so the primary endpoint was not statistically significant; R0 resection was 98% in both groups, while pathological complete response occurred in 7%. In the 2026 NeoCol phase III trial, three-year disease-free survival was 83% after three cycles of CAPOX and 87% after upfront surgery (P = 0.36), again showing no superiority despite downstaging and comparable safety. Together with FOxTROT, these trials argue for selection and shared decision-making rather than routine adoption (20, 21).

Future LACC selection should improve upon imaging stage alone. MMR/MSI is already actionable because conventional chemotherapy benefit may be attenuated in dMMR tumors and prospective immunotherapy studies show marked pathological responses. Radiomics, ctDNA/MRD, sidedness, and broader molecular profiling remain candidate refinements rather than validated preoperative selectors. Accordingly, a patient should not be diverted from timely surgery on the basis of an exploratory biomarker or a radiologic risk label that has not been confirmed in an expert review (22–26).

Neoadjuvant chemotherapy in LACC is therefore classified as Tier 2 selective/conditional use: it may be reasonable for radiologically high-risk, fit patients after MMR/MSI testing and multidisciplinary review, with an explicit reassessment and surgery date. **Table 1** reports the quantitative randomized evidence; **Table 2** specifies the trigger, stopping rule, and boundary.

**Table 1:** Quantitative evidence from pivotal preoperative and response-adapted studies in colorectal cancer.

Study; design	Setting; n	Intervention/primary endpoint	Key efficacy result	Safety/follow-up	Decision boundary	Refs
FOxTROT; phase III	Operable CT-staged T3-4 N0-2 M0 colon cancer; n=1,053	6-week oxaliplatin-fluoropyrimidine before surgery vs postoperative start; 2-year residual/recurrent disease	16.9% vs 21.5%; rate ratio 0.72 (95% CI 0.54-0.98). Histologically complete resection 94% vs 89%.	Obstruction requiring expedited surgery 4.3%; no excess perioperative morbidity.	Supports selective short-course NAC; little apparent benefit in dMMR and no basis for universal use.	(19)
OPTICAL; phase III	LACC; modified ITT n=744	3-month mFOLFOX6/CAPOX then surgery vs upfront surgery; 3-year DFS	82.1% vs 77.5%; HR 0.74 (95% CI 0.54-1.03), primary endpoint not significant. pCR 7%; R0 98% in both.	Median follow-up 48.0 months; secondary OS HR 0.44, interpreted cautiously.	Feasible and downstaging-active, but superiority for DFS was not established.	(20)
NeoCol; phase III	CT-staged LACC; eligible n=248	Three CAPOX cycles vs upfront surgery; 3-year DFS	83% vs 87%; P = 0.36. Adjuvant-treatment indication 59% vs 73%.	Safety and quality of life comparable; 3-year endpoint.	Negative for DFS superiority; reinforces selective rather than routine NAC.	(21)
RAPIDO; phase III	MRI high-risk LARC; eligible n=912	Short-course RT then chemotherapy before TME vs standard CRT-based strategy; disease-related treatment failure	3-year 23.7% vs 30.4%; HR 0.75 (95% CI 0.60-0.95). At 5 years, LRR after R0/R1 was 10% vs 6% (P = 0.027).	Grade >=3 diarrhea 18% vs 9%; median long-term follow-up 5.6 years.	Tier 1 option for MRI high-risk disease; systemic benefit must be weighed against local-control signal.	(27, 28)
PRODIGE 23; phase III	Fit LARC; n=461	Induction mFOLFIRINOX -> CRT -> TME vs CRT -> TME; DFS	7-year DFS 67.6% vs 62.5%; MFS 79.2% vs 72.3%; OS 81.9% vs 76.1%.	During induction, grade 3-4 neutropenia 17% and diarrhea 11%; median follow-up 82.2 months.	Escalation for fit, higher-risk patients; toxicity and generalizability constrain use.	(29, 30)
OPRA; randomized phase II	Stage II/III rectal cancer; n=324	Induction-CRT vs CRT-consolidation TNT; DFS and TME-free survival	5-year DFS 71% vs 69%; TME-free survival 39% vs 54% (P = 0.012). Of 81 regrowths, 94% occurred by 2 years and 99% by 3 years.	Median follow-up 5.1 years; DFS after TME for regrowth vs at restaging 64% vs 64% (P = 0.94).	Consolidation favors organ preservation; W&W requires rigorous assessment, surveillance, and salvage.	(31–33)
PROSPECT; phase III noninferiority	Selected T2N+ or T3 rectal cancer suitable for sphincter-sparing surgery; treated n=1,128	FOLFOX with selective CRT vs routine CRT; DFS	HR 0.92 (90.2% CI 0.74-1.14); 5-year DFS 80.8% vs 78.6%. Only 9.1% received CRT in FOLFOX arm.	Neoadjuvant grade >=3 events 41.0% vs 22.8%; neuropathy vs diarrhea profiles differed; median follow-up 58 months.	Radiation de-escalation only for trial-like eligibility; not for T4 or threatened MRF/CRM.	(34, 35)
NICHE-2; phase II	dMMR colon cancer; enrolled/safety n=115; efficacy n=111	Nivolumab plus ipilimumab before planned surgery; safety and pathological response	Efficacy n=111: pathological response 98%, major response 95%, pCR 68%; no disease recurrence at initial analysis.	Timely surgery 113/115; grade 3-4 immune-related events 4%; median follow-up 26 months.	Strong prospective activity; surgery omission and mature DFS are not established.	(26)
RESET-C; phase II	Stage I-III dMMR colon cancer; enrolled n=85; surgery n=84	Single-cycle pembrolizumab followed by endoscopy/biopsy and planned colectomy; pCR	pCR 44% (37/84; 95% CI 33%-55%); major pathological response 57% (48/84); one recurrence at median follow-up 18.4 months.	Grade 3 adverse events 11% (9/85), including three treatment-related events; no grade 4-5 events; two deaths from surgical complications within 30 days.	Strong activity after one cycle, but colectomy omission is unproven and perioperative risk remains relevant.	(48)
Dostarlimab; single-arm phase II	Locally advanced dMMR rectal cancer; 49 completing therapy in expanded rectal cohort	Six months PD-1 blockade with response-adapted NOM; cCR	All 49 completing patients achieved cCR and entered NOM at report; broader cohort 2-year RFS 92% (95% CI 86%-99%).	Mostly low-grade toxicity; follow-up heterogeneous and single-center specialist program.	Tier 2 molecularly selected NOM; optimal duration, late regrowth, surveillance, and salvage remain uncertain.	(44, 45)
CAIRO5; phase III	Initially unresectable CRLM; modified ITT n=521	Molecular/sidedness-stratified induction; PFS and complete local treatment	Right-sided or RAS/BRAF-mutant: PFS 10.6 vs 9.0 months (HR 0.76); complete local treatment 51% vs 37%. Left-sided WT: response 80% vs 53%, but complete local treatment 58% vs 58%.	Grade >=3 events 76% vs 59% for triplet vs doublet bevacizumab; central review about every 2 months.	Use R0/NED conversion, not response alone, as the endpoint; balance intensity against toxicity.	(18)
TRICE; randomized trial	RAS/BRAF WT initially unresectable CRLM; n=146	Cetuximab-FOLFOXIRI vs cetuximab-FOLFOX; response/conversion	Response 84.7% vs 79.7% (P = 0.42); R0/R1 resection or local treatment 54.2% vs 52.7%; PFS 11.8 vs 13.4 months.	Grade >=3 events 55.9% vs 47.2%; neutropenia 44.1% vs 27.0%.	Triplet chemotherapy did not improve conversion and increased toxicity.	(55)
New EPOC; phase III	Resectable/suboptimally resectable KRAS WT CRLM; n=257	Perioperative chemotherapy +/- cetuximab; PFS/OS	Median OS 81.0 vs 55.4 months; HR 1.45 (95% CI 1.02-2.05), favoring chemotherapy alone. No response/resection advantage.	Median follow-up 66.7 months; grade 3-4 rash 1% vs 16%; five possible treatment-related deaths overall.	Do not extrapolate anti-EGFR activity to routine perioperative treatment of resectable CRLM.	(57)
TransMet; randomized trial	Permanently unresectable, liver-only, BRAF-nonmutated, chemotherapy-responsive CRLM; ITT n=94	Liver transplantation plus chemotherapy vs chemotherapy alone; 5-year OS	ITT 56.6% vs 12.6%; HR 0.37 (95% CI 0.21-0.65). Per protocol 73.3% vs 9.3%.	Median follow-up about 59 months; transplantation adds major surgery, recurrence management, and lifelong immunosuppression.	Exceptional Tier 2 specialist option; narrow selection and organ allocation limit external validity.	(60, 61)

**Table 2:** Scenario-specific decision boundaries, triggers, reassessment points, and evidence tiers.

Clinical scenario	Decision trigger and intent	Recommended pathway	Reassessment/stopping rule	Evidence tier	Boundary	Refs
Radiologically high-risk, resectable LACC	CT T4, threatened margin, bulky nodes or likely multivisceral resection; improve clearance/treat systemic risk	MMR/MSI testing; selective short-course oxaliplatin-fluoropyrimidine; planned colectomy	Early toxicity/obstruction review; restage after planned short course; do not delay resectable surgery	Tier 2: selective/conditional	FOxTROT positive but OPTICAL and NeoCol did not establish DFS superiority; avoid routine use and dMMR extrapolation	(6, 19–21)
MRI high-risk LARC	Threatened MRF/CRM, cT4, EMVI, N2, lateral nodes or high systemic risk; improve local/systemic control	Risk-adapted TNT (RAPIDO-, PRODIGE 23-, or long-course CRT-based strategy)	Composite restaging after TNT; proceed to TME for incomplete response or unsafe margin	Tier 1 when trial eligibility is met	Regimen-specific toxicity and RAPIDO long-term local-control signal; TNT is not automatically organ preservation	(3, 4, 27–30)
Selected lower/intermediate-risk rectal cancer	PROSPECT-like T2N+ or T3, clear MRF/CRM, sphincter-sparing candidate; avoid routine RT	Neoadjuvant FOLFOX with selective CRT or conventional CRT according to MDT	Use protocol response threshold; add CRT for inadequate response	Tier 1, eligibility-dependent	Do not extrapolate to T4, threatened MRF/CRM, bulky or very low tumors outside criteria	(3, 34, 35)
cCR/near-cCR after TNT	Concordant DRE, endoscopy and MRI; preserve rectum	W&W after cCR; short-interval reassessment for near-cCR; TME for incomplete response	DRE/sigmoidoscopy q4 months x2 years then q6 months; MRI q6 months x2 years then annually; annual CT	Tier 2: selective/conditional	Requires adherence, experienced interpretation, rapid salvage access; W&W is active surveillance, not no care	(9, 31, 32, 39–41, 43)
dMMR/MSI-H rectal cancer	Confirmed dMMR/MSI-H; organ-preservation priority	PD-1 blockade in specialist program; response-adapted NOM only after documented cCR	Protocol-defined treatment duration; composite local/systemic reassessment; salvage TME for regrowth or incomplete response	Tier 2: strong prospective, non-definitive	Single-arm evidence; optimal duration, late regrowth and salvage outcomes remain uncertain	(3, 44, 45)
dMMR/MSI-H colon cancer	Confirmed dMMR/MSI-H; pathological downstaging/biologic response	Short-course checkpoint blockade in specialist trial/protocol followed by planned colectomy	Operate on schedule unless protocol specifies otherwise; assess pathology and toxicity	Tier 2: prospective, surgery retained	NICHE-2/RESET-C support response, not colectomy omission; mature DFS is limited	(26, 48)
pMMR/MSS rectal cancer	Research setting and biomarker-selected hypothesis	Checkpoint blockade plus CRT/TNT only in clinical trial	Use trial-defined pCR/cCR and long-term endpoints	Tier 3: investigational	Higher complete-response rates do not establish DFS/OS benefit; PD-L1/immune markers remain exploratory	(46, 49–51)
Initially unresectable but potentially convertible CRLM	No safe complete local plan at baseline; aim for R0/NED	RAS/BRAF- and sidedness-informed induction with anti-EGFR or anti-VEGF context; hepatobiliary MDT	Review about every 2 months; stop at resectability, plateau without gain, progression, liver injury or loss of fitness	Tier 2: conditional, high-level regimen evidence	Response alone is insufficient; protect future liver remnant and account for disappearing lesions and bevacizumab timing	(7, 18, 52, 55, 56)
Resectable CRLM	Safe complete local plan exists; prevent recurrence without losing surgical window	Surgery +/- perioperative chemotherapy; no routine anti-EGFR addition	Coordinate cycles and operative timing; stop for toxicity or threatened resectability	Tier 1 boundary: evidence against routine anti-EGFR addition	New EPOC showed worse OS with cetuximab; conversion evidence must not be imported into resectable disease	(52, 57)
Permanently unresectable liver-only CRLM	Expert panel confirms no resection/ablation pathway; highly selected favorable biology	Consider transplantation plus chemotherapy only in specialized programs	Confirm sustained chemotherapy response, no extrahepatic disease, donor and immunosuppression suitability	Tier 2: specialist/exceptional	Narrow TransMet eligibility, recurrence, immunosuppression, donor scarcity and allocation ethics	(60, 61)

## 5 Total neoadjuvant therapy, response assessment, and organ preservation in rectal cancer

Rectal cancer provides the strongest example of how treatment before surgery can serve different aims. TNT improves delivery of systemic therapy and can be configured for high-risk disease control or for response-adapted organ preservation. These are related but not identical objectives: TNT does not itself equal organ preservation, and W&W is considered only after a composite response assessment and confirmation that intensive surveillance and salvage surgery are feasible.

RAPIDO supports intensification for MRI-defined high-risk LARC but also illustrates why mature follow-up matters. In 912 eligible patients, three-year disease-related treatment failure was 23.7% with short-course radiotherapy followed by chemotherapy and 30.4% with standard chemoradiotherapy-based treatment (hazard ratio 0.75, 95% CI 0.60-0.95). Acute grade 3 or higher diarrhea was more frequent (18% vs 9%). At approximately 5.6 years, distant control remained favorable, but locoregional recurrence after R0/R1 resection was 10% versus 6% (P = 0.027). The regimen is therefore a Tier 1 option for appropriately selected high-risk disease, not a universal template for all rectal cancers (27, 28).

PRODIGE 23 tested induction mFOLFIRINOX before chemoradiotherapy and surgery in fit patients. With 82.2 months of median follow-up, seven-year disease-free survival was 67.6% versus 62.5%, metastasis-free survival 79.2% versus 72.3%, and overall survival 81.9% versus 76.1% in the intensified and control groups, respectively. During induction, grade 3-4 neutropenia and diarrhea occurred in 17% and 11%. These results support escalation for selected fit patients with high systemic risk, while age, performance status, neuropathy risk, and competing morbidity should constrain use (29, 30).

OPRA organized TNT around response-adapted management. Among 324 patients, five-year disease-free survival was similar after induction- versus consolidation-based sequencing (71% vs 69%), whereas five-year TME-free survival favored chemoradiotherapy followed by consolidation chemotherapy (54% vs 39%; P = 0.012). Of 81 local regrowths, 94% occurred within two years and 99% within three years. Disease-free survival after TME performed for regrowth was similar to that after TME at initial restaging, supporting salvageability when regrowth is detected promptly. The clinical implication is not that one sequence is superior for every endpoint, but that consolidation can be favored when organ preservation is a stated priority (31–33).

PROSPECT supplies complementary de-escalation evidence for a narrower, lower-risk population. Among 1,128 treated patients eligible for sphincter-sparing surgery, neoadjuvant FOLFOX with selective chemoradiotherapy was noninferior to routine chemoradiotherapy for disease-free survival (hazard ratio 0.92; five-year disease-free survival 80.8% vs 78.6%), and only 9.1% of the FOLFOX group received preoperative radiation. Grade 3 or higher adverse events during neoadjuvant treatment were more frequent with the longer FOLFOX course (41.0% vs 22.8%), with neuropathy predominating; diarrhea was more prominent with chemoradiotherapy. These findings should not be extrapolated to T4 tumors, threatened MRF/CRM, or other high-risk MRI features (34, 35).

STELLAR and CAO/ARO/AIO-12 add randomized evidence on short-course radiation and sequencing, respectively, while pooled analyses place organ preservation within the wider oncologic context. Taken together, the data support a risk-adapted model: escalation for high-risk MRI features and systemic risk; selective radiation omission only within PROSPECT-like eligibility; and response-adapted organ preservation only when cCR or a carefully followed near-cCR is established (36–38).

A cCR is a clinical inference, not a pathological diagnosis. It should be assigned through concordant DRE, endoscopy, and high-resolution pelvic MRI, preferably after multidisciplinary review. Typical cCR findings are a normal DRE; no visible tumor, ulcer, or nodularity on endoscopy, with a flat white scar and possible telangiectasia; and marked T2 signal normalization or homogeneous fibrosis without restricted diffusion or suspicious nodes. Minor mucosal or MRI abnormalities constitute near-cCR and justify timed reassessment rather than automatic W&W. Routine biopsy is not required to prove cCR because treatment-related fibrosis and sampling error can produce false-negative results (9, 39, 40).

W&W surveillance is most intensive when regrowth is most likely. The OPRA protocol used DRE and flexible sigmoidoscopy every four months for two years and every six months for years 3-5; rectal MRI every six months for two years and annually for years 3-5; and chest, abdominal, and pelvic CT at least annually, with colonoscopy according to guideline schedules. This is a trial-derived framework rather than a universal prescription, but it illustrates the minimum infrastructure required. Suspicious loss of response, endoscopic regrowth, or nodal regrowth should trigger prompt staging and salvage TME rather than prolonged observation (31, 32, 40).

Long-term and real-world evidence supports feasibility while defining the limits. IWWD reported that most local regrowth occurs in the first two years and is predominantly intraluminal, whereas OPRA found 94% of regrowth by two years and equivalent disease-free survival after immediate versus regrowth-triggered TME. In a 2026 single-center cohort, 56 of 148 evaluable patients achieved cCR and 42 entered W&W; at a median 26 months, five recurrences, including three local events, were reported. In the prospective 27-center GRECCAR snapshot, 117 of 457 patients underwent organ preservation, 78% through W&W; 14 W&W regrowths and three recurrences after local excision occurred, mostly during the first year, and salvage surgery was successful. The latter datasets remain limited by selection and short follow-up (32, 41–43).

TNT-induced and immunotherapy-induced cCR share the need for composite assessment, but their evidence bases differ. OPRA provides randomized sequencing and mature regrowth timing after TNT; dMMR immunotherapy data are single-arm, molecularly selected, and less mature for late local failure. W&W outside expert centers is therefore constrained by MRI and endoscopic interpretation, adherence, travel, cost, access to rapid salvage surgery, and the ability to audit outcomes. Patients unable or unwilling to complete intensive surveillance are poor candidates even when an apparent cCR is present (3, 32, 40, 43, 44).

## 6 Neoadjuvant immunotherapy in dMMR/MSI-H and pMMR/MSS colorectal cancer

dMMR/MSI-H CRC illustrates why anatomical stage alone is insufficient. MMR/MSI status should be known before a nonstandard preoperative sequence is chosen. The evidence must nevertheless be separated by site and endpoint: rectal studies primarily address cCR and NOM; colon studies primarily address pathological response before planned colectomy; and pMMR/MSS studies test experimental combinations. These three questions should not be merged under a single claim of immunotherapy sensitivity (3, 26, 44–46).

In locally advanced dMMR rectal cancer, dostarlimab has produced a strong prospective signal. In the expanded single-arm report, all 49 patients in the rectal cohort who completed therapy achieved cCR and entered NOM at the time of reporting; across the broader dMMR cohort, two-year recurrence-free survival was 92% (95% CI 86%-99%). Most adverse events were low grade, but the study remains single-arm and was conducted within a highly specialized program. The six-month protocol used in this study should not be treated as a universally established duration: the optimal number of cycles, stopping criteria, definition and timing of immune-mediated cCR, risk of late local regrowth, surveillance intensity, and outcomes of salvage surgery remain unsettled (44, 45).

For dMMR colon cancer, surgery remains part of the evidentiary pathway. NICHE-2 reported timely surgery in 113 of 115 enrolled patients and grade 3-4 immune-related adverse events in 4%. Among 111 patients in the efficacy analysis, pathological response was 98%, major pathological response 95%, and pathological complete response 68%; no disease recurrence was reported at a median follow-up of 26.2 months, but the primary three-year disease-free survival endpoint was not mature in the initial report. RESET-C enrolled 85 patients; one declined surgery. Among 84 patients who underwent surgery, pathological complete response occurred in 37 (44%) and major pathological response in 48 (57%), with one recurrence at a median follow-up of 18.4 months. Grade 3 adverse events occurred in 9 of 85 patients (11%), including three treatment-related events; no grade 4 or 5 adverse events were reported. Two patients died of surgical complications within 30 days. These safety outcomes reinforce that pathological response does not eliminate perioperative risk. The studies support short-course immunotherapy before colectomy in specialist protocols; they do not establish routine omission of colon resection (26, 47, 48).

The distinction between rectum and colon is clinically consequential. In the rectum, avoiding chemoradiotherapy and TME may preserve bowel, urinary, and sexual function, so a durable cCR can change the treatment endpoint. In colon cancer, imaging and endoscopy cannot validate complete eradication of mural and nodal disease with the same confidence, and the principal prospective endpoint remains pathological response after surgery. Thus, rectal NOM experience should not be extrapolated to colectomy omission.

Immunotherapy-induced cCR also cannot simply inherit every assumption from TNT-induced cCR. Radiation-related fibrosis may complicate MRI and endoscopic interpretation after TNT, whereas immune therapy can produce delayed or heterogeneous regression; in both settings, pCR is unknowable without resection. A patient considered for NOM after immunotherapy should undergo the same composite local assessment and systemic staging used for W&W, with protocol-defined treatment cessation, short-interval reassessment of equivocal findings, and immediate access to salvage TME. Outside expert centers, assay turnaround, radiologic and endoscopic expertise, surveillance adherence, and surgical rescue capacity may be more limiting than drug activity itself (3, 9, 40, 44).

pMMR/MSS rectal cancer remains investigational for immunotherapy. Randomized phase II data suggest that adding sintilimab to chemoradiotherapy can increase complete response, and single-arm studies such as NECTAR report promising pathological response. These response endpoints should not be presented as proof of improved disease-free or overall survival, and exploratory markers such as PD-L1 combined positive score or immune contexture are not validated treatment selectors. Outside a clinical trial, pMMR/MSS status does not justify routine checkpoint blockade before surgery (46, 49–51).

Current immunotherapy evidence therefore supports three boundaries. First, test MMR/MSI early. Second, consider PD-1 blockade and response-adapted NOM for dMMR rectal cancer only within an experienced program that can document cCR and deliver prolonged surveillance (Tier 2). Third, retain planned surgery in dMMR colon pathways and keep pMMR/MSS combinations investigational (Tier 3). **Table 1** reports the key response and follow-up data; **Table 2** separates site-specific decisions.

## 7 Perioperative, conversion, and transplant strategies for colorectal liver metastases

CRLM treatment must be classified by baseline resectability and intent. For resectable disease, surgery with or without perioperative chemotherapy is considered; for borderline or potentially convertible disease, systemic treatment is time-limited and aims to create an R0 resection, ablation, or combined NED strategy; for permanently unresectable liver-only disease, conventional conversion has failed and exceptional local-curative options may be discussed. Response rate is useful only insofar as it changes one of these local-treatment opportunities.

Resectability is a multidisciplinary judgment rather than a lesion-count threshold. Resectable disease permits complete clearance while preserving adequate inflow, outflow, biliary drainage, and future liver remnant. Borderline or potentially convertible disease lacks a safe complete plan at baseline but could become treatable after downsizing, hypertrophy procedures, staged surgery, or combined ablation. Permanently unresectable disease remains unsuitable after expert hepatobiliary review because of anatomy, insufficient remnant, extrahepatic burden, or unfavorable biology. These categories should be recorded at baseline and revisited throughout treatment (52).

In initially unresectable CRLM, primary tumor sidedness and RAS/BRAF status help choose an induction backbone, but treatment intensity must be justified by conversion rather than radiographic response alone. ATOM and BECOME support context-specific anti-EGFR or anti-VEGF approaches. In TRICE, adding irinotecan to cetuximab-FOLFOX increased toxicity without improving objective response (84.7% vs 79.7%) or R0/R1 resection/local treatment (54.2% vs 52.7%). Triplet intensification should therefore not be assumed superior for every RAS/BRAF wild-type patient (53–56).

CAIRO5 operationalized conversion through central expert resectability review and molecular/sidedness stratification. For right-sided or RAS/BRAF-mutant tumors, FOLFOXIRI-bevacizumab improved median progression-free survival over doublet-bevacizumab (10.6 vs 9.0 months; hazard ratio 0.76) and increased complete local treatment from 37% to 51%, at the cost of more grade 3 or higher adverse events (76% vs 59%). For left-sided RAS/BRAF wild-type disease, panitumumab increased response (80% vs 53%) but not complete local treatment (58% in both arms) or progression-free survival. These data show why the MDT endpoint must remain resectability/NED rather than response alone (18).

Negative perioperative evidence is equally important. In New EPOC, adding cetuximab to chemotherapy for resectable CRLM shortened median overall survival from 81.0 to 55.4 months (hazard ratio 1.45, 95% CI 1.02-2.05), without improving response or pathological resection outcomes. Anti-EGFR efficacy in unresectable metastatic disease cannot therefore be extrapolated to routine perioperative treatment of resectable CRLM (57).

Conversion therapy should be reassessed at prespecified short intervals—CAIRO5 used approximately two-monthly panel review—and stopped when a safe complete local plan becomes available, when response plateaus without further technical gain, or when progression, liver injury, or declining fitness makes the original goal unrealistic. Prolonged oxaliplatin or irinotecan exposure may cause sinusoidal injury or steatohepatitis. Disappearing liver metastases should not be assumed eradicated: pretreatment imaging must be retained for operative planning, and lesions may require marking or targeted treatment. Bevacizumab also requires an appropriate preoperative interval to reduce wound-healing risk (18, 52, 58, 59).

TransMet concerns a different population: strictly selected patients aged 18-65 years with resected BRAF-nonmutated primary tumors, no extrahepatic disease, chemotherapy-responsive but permanently unresectable liver-only metastases, and access to transplantation. In 94 randomized patients, five-year overall survival was 56.6% with transplantation plus chemotherapy versus 12.6% with chemotherapy alone (hazard ratio 0.37); the per-protocol estimates were 73.3% versus 9.3%. The magnitude is substantial, but recurrence, lifelong immunosuppression, donor scarcity, and narrow eligibility make transplantation a Tier 2 specialist option, not a general conversion pathway (60, 61).

CRLM management is therefore an MDT algorithm rather than a ranked drug list. It requires an explicit baseline category, molecularly informed induction when conversion is plausible, repeated hepatobiliary review, protection of the future liver remnant, and a recorded stopping rule. **Table 1** presents the quantitative trials; **Table 2** separates resectable, potentially convertible, and permanently unresectable liver-only disease; **Figure 1** displays the reassessment loop.

## 8 Molecular markers and emerging response tools

The rapid expansion of molecular treatment in metastatic CRC creates pressure to move active agents earlier. Such movement is justified only when the marker has been validated for the preoperative decision in question. In this Review, tools are separated into three practical groups: established localized-disease determinants (MMR/MSI and validated anatomical response assessment); context-specific metastatic/conversion determinants (RAS/BRAF status and sidedness); and exploratory tools or advanced-disease targets (HER2-directed therapy, ctDNA/MRD, radiomics, immune signatures, ADCs, bispecific antibodies, and vaccines). This distinction prevents evidence from refractory disease from being mistaken for perioperative benefit.

BRAF V600E and HER2 illustrate this boundary. Encorafenib-cetuximab and HER2-directed regimens, including trastuzumab deruxtecan, are supported in advanced molecularly selected CRC, and these data appropriately inform prognosis, trial referral, and future conversion research. They do not yet define routine preoperative targeted therapy. Statements about these markers should therefore use “may inform” or “supports trial selection” rather than imply that they guide localized-disease treatment (11, 13, 16, 17, 62, 63).

Similarly, ADCs, bispecific antibodies, and vaccines remain Tier 3 for preoperative CRC. Early-phase activity in advanced CEACAM5-, EGFR-, VEGF-, KRAS-, or immune-selected disease establishes biological plausibility, not surgical benefit. Prospective perioperative studies must demonstrate safety around surgery, meaningful pathological or conversion endpoints, and durable disease control before these agents enter routine algorithms (64–68).

ctDNA/MRD is prognostic after curative-intent treatment and has supported randomized adjuvant de-escalation in stage II colon cancer, but its preoperative role remains adjunctive. Assays differ in tissue dependence, limit of detection, sampling schedule, turnaround time, and false-negative risk. A positive result may justify closer evaluation or trial enrollment; a negative result must not override suspicious MRI, endoscopy, DRE, or a resectable lesion. At present, ctDNA alone should not determine W&W, omit surgery, or trigger routine preoperative escalation (69–72).

Radiomics and artificial intelligence face a related translation gap. Retrospective models can predict pathological response or recurrence, but scanner protocols, segmentation, feature extraction, small training sets, and limited external validation reduce reproducibility. Their current role is hypothesis generation or decision support within validation studies, not independent treatment allocation (73–75).

Implementation constraints must be included in any claim of molecularly informed care. Comprehensive profiling is not equally available across regions; tissue quantity, payer coverage, cost-effectiveness, and assay turnaround may make results unavailable before a surgical decision. ctDNA platforms are not interchangeable, and repeated testing adds cost. High-quality pelvic MRI, expert radiology review, endoscopy, and W&W surveillance also require infrastructure that may be less accessible than the drugs themselves. These limitations should determine whether a pathway is feasible locally, not merely whether it is biologically attractive.

**Table 3** therefore reports analytical or clinical validation, guideline incorporation, prospective intervention data, potential escalation/de-escalation role, current applicability, and implementation limitations for each marker or tool. The evidentiary standard should rise as a technology moves earlier in the treatment course and begins to replace surgery or radiotherapy.

**Table 3:** Evidence maturity and current clinical applicability of biomarkers and dynamic tools.

Marker/tool	Validation and guideline position	Prospective intervention data	Potential decision role	Current applicability	Access/technical limitations	Refs
MMR/MSI	Analytically established and recommended early in CRC; direct localized treatment relevance	Dostarlimab, NICHE-2 and RESET-C provide prospective site-specific evidence	Select immunotherapy-first approaches; possible rectal NOM after cCR	Routine testing; treatment use is context-specific: early testing is guideline-aligned, whereas surgery deferral after rectal cCR remains Tier 2	Assay discordance/rare heterogeneity; specialist response assessment and long surveillance needed	(3, 6, 26, 44, 45, 48)
RAS/BRAF status and primary sidedness	Established predictors for metastatic systemic therapy; relevant to CRLM induction	CAIRO5, TRICE, BECOME and ATOM	Choose anti-EGFR vs anti-VEGF context and intensity in conversion planning	Routine in metastatic/CRLM work-up; not a stand-alone perioperative indication	Turnaround and tissue adequacy; biology does not replace resectability review; New EPOC boundary	(7, 18, 54–57)
HER2	Actionable in advanced molecularly selected CRC; not established for localized preoperative care	Prospective anti-HER2/ADC data are mainly refractory or metastatic	Trial referral and future conversion research	Tier 3 for routine preoperative decisions	Testing methods/cutoffs and access vary; perioperative efficacy and surgical safety unproven	(14, 16, 17)
Composite rectal response (DRE, endoscopy, MRI)	Clinically validated and incorporated into specialist W&W pathways	OPRA and prospective/registry organ-preservation datasets	Select W&W, monitor near-cCR, detect salvageable regrowth	Tier 1 assessment tools; Tier 2 decision to defer surgery	Operator dependence; MRI/endoscopic expertise, adherence and salvage access required	(9, 31, 32, 39–41, 43)
ctDNA/MRD	Strong prognostic validity after curative-intent therapy; platform-specific	Randomized adjuvant de-escalation data, but little direct preoperative intervention evidence	Research-based risk refinement, surveillance and future escalation/de-escalation trials	Adjunctive/Tier 3 for surgery omission or preoperative allocation	Tumor-informed vs agnostic differences; sensitivity, timing, tissue dependence, cost and turnaround	(69–72)
Radiomics/AI	Heterogeneous retrospective validation; no routine treatment-allocation guideline role	No definitive prospective intervention evidence for most CRC preoperative decisions	Future response prediction and risk stratification	Tier 3/investigational	Scanner and protocol variation, segmentation, feature instability, small datasets, weak external validation	(73–75)
Immune contexture, PD-L1 CPS, POLE/POLD1	Biologically relevant; predictive role in pMMR neoadjuvant treatment not established	Exploratory biomarker analyses and early prospective combinations	Trial enrichment for pMMR/MSS immunotherapy	Tier 3/investigational	Assay and cutoff variability; response association does not prove long-term benefit	(46, 49, 51)
ADCs, bispecific antibodies, vaccines	Agent-specific activity in advanced disease; no established preoperative guideline role	Predominantly phase I/II advanced-disease studies	Future molecularly selected conversion or perioperative trials	Tier 3/investigational	Cost, toxicity, target heterogeneity, perioperative safety and lack of durable surgical endpoints	(16, 64–68)

The practical rule is simple: a biomarker should change a preoperative decision only when the clinical context and consequence have been prospectively supported. MMR/MSI meets this threshold in selected localized settings; RAS/BRAF and sidedness are relevant to CRLM induction choices; most other tools remain supportive or investigational. **Figure 2** uses the same Tier 1-3 categories so that visual prominence does not imply equal maturity.

## 9 Discussion

The current shift is not simply toward more treatment before surgery. It is toward explicit matching of disease state, treatment intent, evidence level, and response. The revised framework therefore separates standard high-level pathways from selective specialist strategies and investigational concepts, and it attaches a trigger, reassessment point, and stopping rule to each major scenario.

Several conclusions are sufficiently mature for practice. High-risk LARC is the best-established setting for TNT, while PROSPECT-like patients may avoid routine radiation. MMR/MSI should be obtained early because it can alter localized treatment. In LACC, neoadjuvant chemotherapy remains selective in light of mixed phase III results. In CRLM, sidedness and RAS/BRAF status inform induction, but repeated expert resectability review—not maximum radiographic response—determines whether conversion has succeeded.

Implementation will be less uniform than trial efficacy. A molecularly informed pathway depends on timely pathology and molecular testing, high-quality pelvic MRI, hepatobiliary expertise, coordinated medical and radiation oncology, and a surveillance system capable of detecting regrowth while salvage remains feasible. Cost, payer coverage, assay turnaround, travel burden, and adherence can convert an otherwise reasonable W&W or molecular pathway into an unsafe one. Centers without these resources should favor an evidence-based conventional pathway or refer the patient rather than reproduce only part of a specialist protocol.

Terminology is part of clinical safety. Neoadjuvant therapy assumes a resectable tumor and planned surgery; TNT includes both systemic chemotherapy and radiotherapy; perioperative treatment spans an operation; conversion therapy starts from unresectability; organ preservation is an outcome; NOM is the decision to defer surgery; and W&W is the structured surveillance used after that decision. cCR is a clinical composite and must not be conflated with pCR, which is a pathological endpoint available only after resection.

Important questions remain unresolved. LACC requires better selectors for benefit. Rectal organ preservation requires standardized near-cCR handling, surveillance outside expert centers, patient-reported outcomes, and long-term regrowth and salvage data. dMMR rectal cancer requires evidence on immunotherapy duration, stopping criteria, late recurrence, and rescue surgery. dMMR colon cancer requires trials before colectomy omission can be considered. CRLM requires reproducible resectability definitions, prospective stopping rules, and better integration of liver injury and disappearing lesions. ctDNA and radiomics require intervention trials showing that acting on a result improves outcomes.

For these reasons, the frameworks proposed in this Review should not be read as prescriptive protocols. They are intended to support structured MDT discussion, while leaving room for institutional expertise, patient preference, local diagnostic capacity, and the availability of reliable surveillance and salvage pathways.

This Review has limitations. It is a structured narrative Review rather than a systematic review or meta-analysis, and no formal risk-of-bias tool or quantitative pooling was used. The original database export was not retained, so the reproducible update strategy is reported without reconstructing a retrospective PRISMA flow. The core selection, full-source verification, evidence extraction, and drafting were led by WH, with revision-stage literature searching and evidence organization by YL and senior intellectual adjudication by HC and MH; residual qualitative selection bias remains. We mitigated this by retaining negative trials, mature follow-up that altered interpretation, and explicit evidence tiers. Because immunotherapy, organ preservation, and biomarkers are changing rapidly, the framework should be updated when new phase II/III results or guideline changes alter a tier, trigger, or stopping rule.

Trial endpoints must also match the decision. In rectal cancer, cCR, TME-free survival, local regrowth, salvageability, stoma-free survival, function, and quality of life may be as important as pCR. In CRLM, response must be linked to R0/NED treatment, liver toxicity, recurrence, and survival. In immunotherapy, early response should be interpreted with durability and the safety of any surgery-omission strategy. **Table 1** therefore reports quantitative outcomes and follow-up rather than relying on adjectives such as “remarkable” or “durable”.

Future research should prioritize prospective biomarker-driven trials that explicitly test escalation and de-escalation. dMMR/MSI-H trials should define immunotherapy duration, surgery timing, and surveillance criteria. pMMR/MSS immunotherapy trials should avoid unselected adoption and should include robust translational biomarkers. CRLM trials should standardize resectability definitions and MDT reassessment schedules. ctDNA/MRD trials should test whether changing management based on a molecular signal improves patient outcomes, not simply whether the signal predicts recurrence. Emerging targeted therapies, ADCs, bispecific antibodies, and vaccines should be evaluated in molecularly selected preoperative or conversion settings only when perioperative safety and long-term endpoints are built into the design.

The contribution of this Review is a common decision grammar rather than a single protocol. Each pathway is defined by disease state, intent, evidence tier, trigger, reassessment, and boundary. This makes escalation, de-escalation, organ preservation, conversion therapy, and emerging molecular approaches comparable without suggesting that they are interchangeable or equally mature.

## 10 Conclusions

Preoperative and response-adapted management of CRC is becoming more individualized, but its components have unequal evidentiary maturity. TNT is established for selected LARC, W&W is conditional on a documented cCR and intensive surveillance, neoadjuvant chemotherapy for LACC remains selective, dMMR immunotherapy is highly active but site-specific, and CRLM conversion requires repeated expert assessment.

High-quality imaging and early molecular testing should precede any nonstandard sequence. MMR/MSI can directly alter localized treatment; RAS/BRAF status and sidedness inform CRLM induction; HER2 is mainly relevant to advanced-disease therapy and trials; and ctDNA, radiomics, and most emerging platforms remain supportive or investigational. The decision to intensify, de-escalate, defer surgery, or proceed to a local-curative intervention should be tied to a prespecified endpoint and salvage plan.

Earlier treatment is not automatically better treatment. The clinically useful question is whether a defined patient, in a defined setting, is more likely to benefit than to be harmed when therapy is moved earlier or surgery is deferred. A transparent evidence tier and reassessment rule make that judgment more reproducible and more honest.
